# Crystal Structures of Influenza A Virus Matrix Protein M1: Variations on a Theme

**DOI:** 10.1371/journal.pone.0109510

**Published:** 2014-10-08

**Authors:** Martin K. Safo, Faik N. Musayev, Philip D. Mosier, Qibing Zhou, Hang Xie, Umesh R. Desai

**Affiliations:** 1 Department of Medicinal Chemistry, School of Pharmacy and Institute for Structural Biology and Drug Discovery, Virginia Commonwealth University, Richmond, Virginia, United States of America; 2 Institute of Materia Medica, College of Life Science and Technology, Huazhong University of Science and Technology, Wuhan, Hubei, P. R. China; 3 Division of Viral Products, Office of Vaccines Research and Review, Center for Biologics Evaluation and Research, United States Food and Drug Administration, Bethesda, Maryland, United States of America; Centro de Biología Molecular Severo Ochoa (CSIC-UAM), Spain

## Abstract

Matrix protein 1 (M1) of the influenza A virus plays multiple roles in virion assembly and infection. Interest in the pH dependence of M1's multiple functions led us to study the effect of subtle pH changes on M1 structure, resulting in the elucidation of a unique low-pH crystal structure of the N^1-165^-domain of A/WSN/33 (H1N1) M1 that has never been reported. Although the 2.2 Å crystal structure of M1 N-terminus shows a dimer with the two monomers interacting in a face-to-face fashion at low pH as observed earlier, a 44° rotation of the second monomer has led to a significantly different dimer interface that possibly affects dimer stability. More importantly, while one of the monomers is fully defined, the N-terminal half of the second monomer shows considerable disorder that appears inherent in the protein and is potentially physiologically relevant. Such disorder has not been observed in any other previously reported structure at either low or high pH conditions, despite similar crystallization pH conditions. By comparing our novel N^1-165^-domain structure with other low-pH or neutral-pH M1 structures, it appears that M1 can energetically access different monomer and dimer conformations, as well as oligomeric states, with varying degree of similarities. The study reported here provides further insights into M1 oligomerization that may be essential for viral propagation and infectivity.

## Introduction

The matrix protein 1 (M1) of the influenza A virus is a 252-amino acid protein [1], comprised of an N-terminal domain (165 amino acids; N^1–165^-domain) and a C-terminal domain (87 amino acids; C^166–252^-domain), and plays an essential role in structural integrity, replication and budding of the virus. In the mature virion, M1 stabilizes the two major viral surface glycoproteins hemagglutinin (HA) and neuraminidase (NA) by forming a matrix layer underneath the lipid bilayer of the viral envelope [2]. M1 also directly interacts with viral ribonucleoprotein (vRNP) consisting of viral RNA (vRNA), RNA polymerases and RNA-binding nucleoprotein (NP) and mediates the import of vRNP for vRNA synthesis in the nucleus of infected cells. Most of these functions are believed to be executed via the 18 kDa N^1–165^-domain of M1 involving the positively charged nuclear localization signal (NLS) motif ^101^RKLKR^105^ and other basic residues adjacent to the NLS [3]–[11].

Previous structural studies on the truncated N^1–165^-domain have focused on either low pH (pH∼4.5) [4], [12], a condition similar to endosome acidification after virus particles are internalized [13], or neutral pH resembling the cytoplasmic environment at which virus particles form [14], [15]. Despite both structures showing identical monomer folds consisting of two 4-helix bundles that are connected by another helix-containing segment, the low-pH structures show a dimeric structure in contrast to the loosely arranged monomeric structures with very different monomer–monomer arrangements observed at neutral pH, resulting in significantly different models proposed for M1 oligomerization and assembly [12], [14].

In the early stages of virus replication, HA of internalized virions undergoes major conformational changes in the acidified endosome (pH∼5) for membrane fusion and subsequent release of vRNA into the cytoplasm [16]. The pH inside the virions also drops by protons pumped through the transmembrane M2 ion channel that directly interacts with M1 [17]. Using cryo-electron tomography, Fontana et al. [18] have demonstrated that the HA spikes on the surface of virions become disorganized at pH 4.9, which is accompanied by the disappearance of the M1 matrix layer. Exposure of virions to low pH also causes the conformational changes that disrupt M1-M1 interactions and render the M1 layer thinner, resulting in the relaxation of the M1 layer from the viral envelop [19]. The disruption of the M1 matrix layer in the postfusion stage is believed to be associated with the release of vRNP [20]. In addition to interacting with viral components, M1 is also associated with the host cellular membrane in the later stages of virus replication for assembly and budding. The association of M1 with the host cellular membrane is also likely influenced by the physiological pH of respiratory tract. Taken together, these pieces of evidence allow us to hypothesize that M1 might have multiple conformational transitions with certain flexibility in its oligomeric structures corresponding to its multiple functions and complex interactions with other viral and host components. The current crystal structures of the N^1–165^-domain at either low or neutral pH [4], [12], [14], [15] may not adequately address all potential multi-conformational changes of M1 when it shuttles between the nucleus and the host cellular membrane for virus propagation. We are particularly interested in how subtle pH changes affect the structure of the N^1-165^-domain and how these structural changes impact multiple functions of M1. In the present study, we report a unique low-pH crystal structure of the M1 N^1-165^-domain of A/WSN/33 (H1N1) that shows a significant rotation (44–48°) of one of the monomers, resulting in a distinguishing dimer arrangement from previously reported low-pH M1 structures (*vide infra*). This novel low-pH M1 structure also shows a region of major disorder in one of the monomers. We have compared this novel M1 N^1-165^-domain structure with several previously solved M1 structures for insights into M1 oligomerization, and described how the partially unfolded M1 structure may be physiologically important for viral function.

## Materials and Methods

### Protein production

The N-terminal domain (1–165 aa) of M1 was subcloned into pET30b (Novagen) from a pHW2000 plasmid expressing the full-length A/WSN/33 M1 [10] at the cloning sites of Nde1+Xho1. The new plasmid pET30b-M1wt-1–165 with a His-tag at the N-terminus (5′-primer: actagccatatgcaccatcatcatcatcatagtcttctaaccgaggtc; 3′-primer: tctgatctcgagctacatttgcctatgagaccgatg) was expressed in *E. coli* strain Nico21(DE3) (NEB). The His-tagged N^1–165^-domain of M1 was further purified by affinity chromatography in combination with FPLC columns (Excellgen, MD) with the purity>90% by SDS-PAGE analysis. The recombinant protein was kept in 55 mM KH_2_PO_4_/K_2_HPO_4_, 0.2 M NaCl, 2 mM TCEP buffer at pH 4.0.

### Crystallization and data collection

Crystals of the N^1–165^-domain of M1 were obtained by the sitting-drop vapor diffusion method with 5 µL drops consisting of a 1:1 ratio of protein:reservoir solution equilibrated against 600 µL of reservoir solution at 20°C. Protein concentration varied from 9 to 25.8 mg/ml in 55 mM KH_2_PO_4_/K_2_HPO_4_/H_3_PO_4_, 0.2 M NaCl, 2 mM TCEP buffer, pH 4.0. The best crystals were obtained at 25.8 mg/ml protein concentration with a reservoir solution containing 15% PEG-3K in 75 mM Na citrate buffer, pH 5.5.

For X-ray data collection, crystals were cryoprotected in their mother liquid solution supplemented with 25% PEG-3K before flash cooling in liquid nitrogen stream. The X-ray data set was obtained at 100 K on an R-axis IV++ image plate detector using CuKα X-ray (λ = 1.5417) from a Rigaku Micro-MaxTM-007 X-ray source equipped with Varimax confocal optics operating at 40 kV and 20 mA (Rigaku, The Woodlands, TX). Crystals diffracted to 2.2 Å resolution, and the data set was processed and scaled with Rigaku D*TREK software.

### Structure determination

Structure determination was carried out by molecular replacement with the program AutoMR Phenix v.1.8 [21]. Using the monomeric structure of the truncated N^1–165^-domain of M1 (Influenza A/PR/8/34) with PDB entry code 1EA3 or 1AA7 resulted in a solution of two monomers per asymmetric unit; LLG = 1043.3, Z score = 18.0, R-value = 48.8. Initial refinement showed extensive disorder at the first 80 N-terminal residues of monomer B, while monomer A was well-defined except for the loop region residues 69–76. Only residues 2–16, 20–27, 45–71, and 75–156 could be accounted for in monomer B, while monomer A included residues 1–158. Final refinement with 158 water molecules resulted in R_work_/R_free_ of 22.6/26.4. Data collection and refinement statistics are summarized in Table 1.

**Table 1:** Data collection and refinement statistics of Safo-4PUS-pH4.7.

**Data collection statistics**	
Space group	*P2_1_*
Cell dimensions (Å)	a = 54.30, b = 37.35, c = 94.97
Resolution (Å)	29.09–2.20 (2.28–2.20)
No. of measured reflections	66735
Unique reflections	19111 (1858)
Redundancy	3.49 (3.28)
I/σI	15.2 (4.7)
Completeness (%)	99.1 (99.1)
R_merge_ (%)^a^	4.5 (23.7)
**Structure refinement**	
Resolution limit (Å)	29.09–2.20 (2.28–2.20)
No. of reflections	18978 (1831)
R_work_ (%)	22.6 (28.3)
R_free_ (%)^b^	26.4 (31.6)
R.m.s.d. standard geometry	
Bond lengths (Å)	0.009
Bond angles	1.4°
Dihedral angles (%)	
Most favored regions	82.3
Allowed regions	16.8
Average B-factors (Å^2^)	
All atoms	56
Protein alone	57
Water	54

a R_mege_ = Σ_hkl_Σ_i_/I_hkli_–<I_hkli_>/Σ_hkl_Σ_i_<I_hkli_>.

b R_free_ was calculated with 5% excluded reflection from the refinement.

## Results

### Crystallization and structure of the N-terminal domain of M1

**Figure 1:**
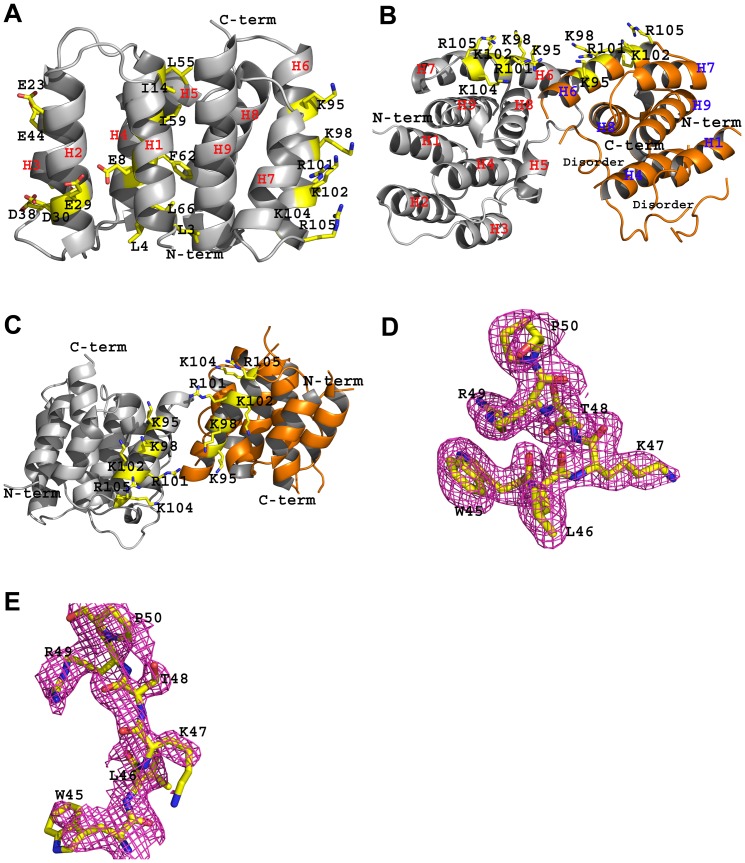
Structure of truncated N^1-165^ domain of M1 (PDB code 4PUS) at pH 4.7. A. Monomer A of 4PUS. The N-terminal and C-terminal domains are shown in grey ribbons. Shown in yellow sticks are the basic NLS motif residues (Arg101, Lys102, Lys104, Arg105), Lys95 and Lys98 located on the right face of the molecules; the negatively charged residues Glu8, Glu23, Glu29, Asp30, Asp38, and Glu44 located on the left face of the molecule; and representative hydrophobic core residues on H1 and H4 buried between the N-terminal and C-terminal domains. For clarity not all residues of interest are shown. The nine helices are labelled **B**. Dimeric structure showing the positively charged residues including the NLS (yellow sticks) on the surface of the molecule. Monomers A and B are colored in grey and orange, respectively. Note the disorder in the N-terminal domain of monomer B. **C**. Same as figure B, but rotated by ∼90°. **D and E**. Final refined 2Fo-Fc electron density maps (contoured at 0.9α) of analogous regions (residues 45–50) of monomers A and B, respectively. The region in monomer B is clearly disordered.

We have crystallized the truncated N^1–165^-domain of M1 using low salt and low pH (∼4.7) conditions in the space group *P2_1_* with unit-cell parameters a = 54.30, b = 37.35, c = 94.97 Å and β = 102.4°, and one dimer (monomers A and B) per asymmetric unit (Fig. 1A–C). Structure determination by molecular replacement using other reported M1 structures, including 1AA7 or 1EA3 was only successful with a monomer rather than a dimer, suggesting different oligomeric arrangements compared to either known structure. The structure has been refined to a R_work_/R_free_ of 22.6/26.4, and deposited in the PDB with ID code of 4PUS, which will subsequently be used to refer to this structure.

The entire chain of monomer A of 4PUS with the exception of residues 69–76 that show weak density is relatively well-defined with essentially the same secondary structures as the previously reported M1 structures, 1AA7 or 1EA3. This consists of nine α-helices, including an N-terminal domain containing helices H1 (3–12), H2 (19–32), H3 (39–48), and H4 (54–67) connected to a C-terminal domain containing helices H6 (90–105), H7 (109–116), H8 (121–132) and H9 (140–157) by a helix-containing H5 (78–84) linker (Fig. 1A). As depicted in the figure, one face of the monomer has a continuous patch of positively charged amino acids that include the basic NLS motif residues (Arg101, Lys102, Lys104, Arg105), as well as Arg134, Lys95, and Lys98. In contrast, the opposite face of M1 is negatively charged and includes the residues Glu8, Glu23, Glu29, Asp30, Asp38 and Glu44 (Fig. 1A). The patch of positively and negatively charged residues has been implicated in several viral protein binding events as well as in M1 oligomerization [3]–[5], [7], [9]–[11].

Monomer B shows significant disorder at the N-terminal domain containing residues 1–81 (H1–H5), and as a result residues 1, 17-19, 28–44, 72–74 could not be modelled (Fig. 1B and 1C). Figure 1 (D and E) shows analogous electron density maps of monomers A and B that illustrate a typical disorder in monomer B. Even the N-terminal domain residues that were included in the model show significantly higher than average B-factors of 92 Å^2^ compared to 67 Å^2^ for the C-terminal domain residues 82–156; similarly, the average B-factor for the entire B-chain was 78 Å^2^ compared to 38 Å^2^ for the entire A-chain. Importantly, helices H1–H5 seem to have unwound, with H2, H3 and H5 becoming unstructured (Fig. 1B and 1C). No other M1 structure shows such an extensive disorder, although residues 69-77 are known to be weak or disordered in other M1 structures. The reason behind the extensive disorder in 4PUS is not apparent because some of the disordered areas are actually close to crystal and/or molecular contacts. Moreover, analogous regions in monomer A that lack crystal contacts are ordered. This could also be said of 1AA7 and 1EA3 which show such regions to be relatively well-ordered and resolved even though some of these areas are exposed to the bulk solvent. Another M1 structure from an independently grown crystal using the same crystallization condition as 4PUS (same space group and similar cell constants) showed similar extensive disorder. Since the 2.4 Å structure was indistinguishable from the 2.2 Å 4PUS, the refinement was terminated. These observations suggest an inherent disorder in the protein that maybe physiologically relevant.

Superposition of monomers A and B of 4PUS resulted in an RMSD of 2.2 Å (using all but eight Cα residues present in monomer B). This compares to an RMSD of 0.5 Å when only the C-terminal Cα residues (81–155) are superposed suggesting significant structural perturbation at the first 80 N-terminal residues. Nonetheless, the relative positions of the N- and C-terminal domains of the two subunits do not change substantially and the significant structural differences are mainly due to the disorder of monomer B's N-terminal domain. The N- and C-terminal domains are held together by strong but buried hydrophobic interactions from H1 and H4 of the N-domain to H8 and H9 of the C-domain. Sha & Luo have proposed a possible conformational change in M1 that could expose the buried hydrophobic residues (Leu3, Leu4, Val7, Tyr10, Val11, Ile14, Pro54, Leu55, Ile59, Phe62, Val63 and Leu66; see Fig. 1A) on H1 and H4 for membrane binding [12]. Although the unwinding of the N-terminal domain has not led to exposure of the H1 and H4 hydrophobic residues, this observation for the first time may indicate beginning of a domain rotation that exposes these residues as proposed by Sha & Luo [12].

Monomers A and B are related by a non-crystallographic 2-fold (perpendicular to H6) symmetry to form a dimer; placing the monomers in a face-to-face fashion that brings the two basic faces adjacent to each (Fig. 1B and 1C). The solvent accessible buried surface area, calculated using PDBePISA is 1303 Å^2^ [22], [23]. The dimerization is maintained primarily by hydrophobic interactions between the symmetry-related helices H6 and to a lesser extent between helices H9 and loops L9, augmented by hydrogen-bond interactions involving Asn82, Arg101 and Asn133 of monomer A to Asn82, Asp94 and Gly88 of Monomer B (Table 2).

**Table 2:** Hydrogen bond interactions (<3.8 Å) and solvent accessible buried surface area of the monomer–monomer interface of M1 structures.

Monomer A	Monomer B	Distance (Å)	Buried Surface Area (Å)
**4PUS**			
Asn82(OD1)	Asn82(ND1)	3.5	
Asn82(ND2)	Asn82(OD1)	3.2	
Asn87(O)	Asn133(ND2)	3.6	1303
Asn133(ND2)	Gly88(O)	2.6	
Arg101(NH2)	Asp94(OD1)	3.5	
**1AA7**			
Gln75(OE1)	Arg78(N)	2.9	
Arg78(N)	Gln75(OE1)	3.0	
Arg76(O)	Gln75(NE2)	3.2	
Gln81(OE1)	Arg134(NE)	3.1	
Gly88(O)	Tyr100(OH)	2.9	
Tyr100(OH)	Gly88(O)	2.7	
Arg101(NH2)	Asn91(OD1)	3.0	2183
Asn91(OD1)	Arg101(NH1)	3.4	
Asn133(O)	Asn85(ND2)	3.1	
Asn85(ND2)	Arg134(O)	3.1	
Arg134(O)	Asn85(ND2)	3.2	
Gln75(NE2)	Arg77(NH1)	3.4	
Asn133(ND2)	Asn133(O)	3.6	
**3MD2**			
Asn85(OD1)	Arg134(NH1)	2.6	
Arg134(NH1)	Asn85(ND2)	3.3	
Asn85(ND2)	Arg134(NH1)	3.3	
Asn133(O)	Arg78(NH2)	2.6	
Arg78(NH2)	Asn133(O)	2.7	
Gln81(NE2)	Gln81(OE1)	3.5	1459
Lys98(NZ)	Asp94(OD2)	3.8	
Asp94(OD2)	Lys98(NZ)	3.7	
Lys104(NZ)	Asp89(OD1)	3.8	
Asp89(OD1)	Lys104(NZ)	3.8	
**1EA3**			
Asp94(OD2)	Lys21(NZ)	3.2	
Tyr100(OH)	Glu29(OE1)	3.5	
Tyr100(OH)	Glu29(OE2)	3.7	
Arg134(NH1)	Asp30(OD1)	2.7	
Arg134(NH2)	Asp30(OD2)	2.5	896
Arg101(NH2)	Glu8(OE1)	3.4	
Arg101(NH2)	Glu8(OE2)	3.4	
Lys104(NZ)	Glu29(OE2)	3.1	
Asp94(OD2)	Ser17(OG)	3.1	
Asp89(OD1)	Asn36(ND1)	3.5	
**2Z16**			
Ser118(O)	Thr37(N)	2.9	
Ser118(O)	Thr37(OG1)	3.2	
Ser118(OG)	Thr37(OG1)	3.3	423
Arg95(NH1)	Thr37(OG1)	3.1	
Arg95(NH2)	Thr37(OG1)	3.3	

### Structure comparison with other M1 structures

**Figure 2:**
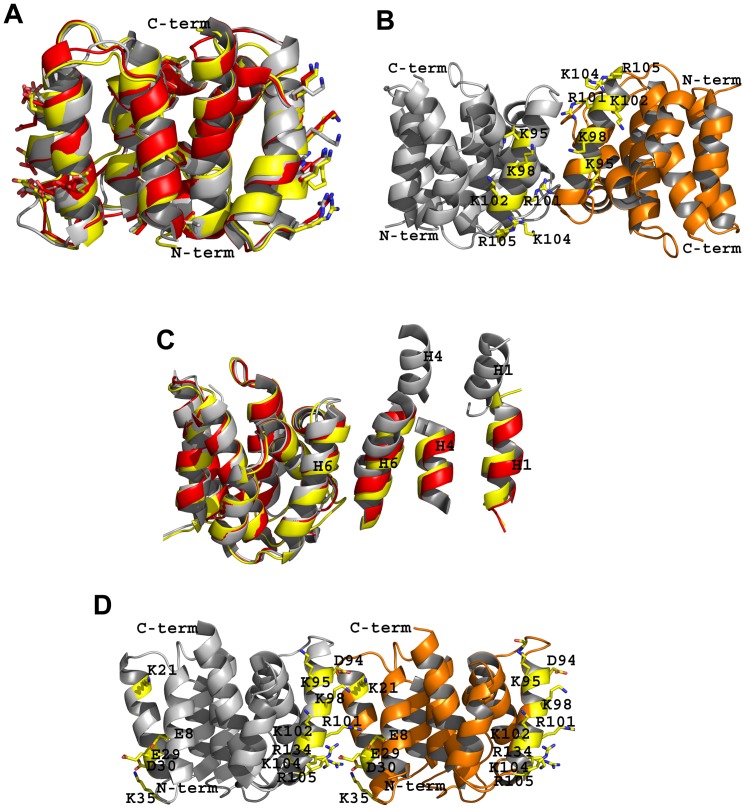
Comparison of 4PUS with other truncated N^1-165^ domain M1 structures (in ribbon diagrams). Unless noted otherwise, monomers A and B are colored grey and orange, respectively. **A**. Superposition of monomers A of 4PUS (grey), 1AA7 (yellow), and 1EA3 (red). **B**. Dimeric structure of 1AA7 showing face-to-face interaction between the two monomers. **C**. Relative positions of the non-superposed monomers B of 4PUS (grey), 1AA7 (yellow) and 3MD2 (red) after superposition of the corresponding monomers A. For clarity, only parts of the non-superposed monomers B are shown. **D**. Two monomers of 1EA3 interacting in a face-to-back fashion with the positively charged residues facing the negatively charged residues. For clarity not all positive or negative residues at the monomer faces are shown.

The M1 N^1–165^-domain structure has previously been crystallized by several workers at both low and neutral pHs. The low-pH structures include our reporting structure, 4PUS (pH∼4.7), and 1AA7 (pH∼4.3) reported by Sha & Luo [12]. A neutral-pH structure 1EA3 (pH∼7.5) has been reported by Arzt et al. [14]. Two other structures 3MD2 and 2Z16 are also available although the pHs of crystallization are unknown. The M1 structures contain two molecules of M1 in the asymmetric unit with the exception of 3MD2 which has four molecules. Unlike 4PUS, it appears none of the proteins used to crystallize the other reported M1 structures were expressed with a histag, although we did not observe any of the N-termini tagged histidines due to apparent disorder. Least-squares superpositions of monomers A of 4PUS and the other M1 structures resulted in RMSDs of ∼0.8 Å, and show similar monomeric structures (Fig. 2A). The RMSDs increased slightly to 1.0–1.3 Å when all Cα residues (4–155) are used for the comparison, which is primarily due to differences in the positions of helix H1 and the flexible loops.

As discussed above and also previously reported [4], [12], [14], [15], despite similar monomeric conformations, the monomer–monomer associations among the structures are quite different. Similar to the low-pH 4PUS (Fig. 1C), the low-pH 1AA7, as well as 3MD2 also dimerize in a face-to-face fashion (Fig. 2B). Nonetheless, there is a significant difference in the relative arrangements of the monomers forming the dimers, which can be quantified by first superimposing the monomers A of the structures, and determining the positional difference in their respective monomers B. Comparison of 4PUS with 1AA7 or 3MD2 shows that a screw rotation angle/translation of 43.8°/0.3 Å or 47.6°/0.5 Å is needed to superpose the monomers B, respectively. Interestingly, the dimer arrangement between 1AA7 and 3MD2 are very similar, requiring only a rotation/translation of 5.3°/1.2 Å to superpose the monomers B. Figure 2C shows the different positions of monomers B (only parts of monomer B shown for clarity) after superposition of the respective monomers A of 4PUS, 1AA7 and 3MD2, and clearly illustrates a gradual sliding of the dimer interface, as well as rotation of the monomer as we move from one structure to another. The different dimer arrangements have resulted in major differences in monomer–monomer interface geometry, interactions and buried surface (Table 2). For example, the inter-subunit hydrogen-bond interactions and hydrophobic interactions are much more extensive in 1AA7, followed by 3MD2 and lastly 4PUS that are also reflected in the buried surface area: 2183 Å^2^ in 1AA7, 1459 Å^2^ in 3MD2 and 1303 Å^2^ in 4PUS (Table 2). We should note that even though both 4PUS and 3MD2 have significantly reduced buried solvent accessible surface areas, they fall within the definition of a dimer [24], [25]. It appears that the fulcrum of the subunit rotation is centered about residues Asn85, Gly86, Asn87, Asn133 and Arg134, although specific interactions involving these residues are different, especially between 4PUS and the other two structures.

Interestingly, unlike the low-pH structures, monomers A and B in the neutral-pH structure of 1EA3 are arranged in a face-to-back fashion, where the continuous patch of positive residues at one side of monomer A (Arg101, Lys102, Lys104, Arg105, Arg134, Lys98, and Lys95) plus Asp94 interact with the patch of negative residues (Glu8, Glu23, Glu29, and Asp30) plus Lys35 at the back side of monomer B (Fig. 2D; Table 2). Very few of the contacts between the monomers are hydrophobic, which may explain the smaller buried surface area (896 Å^2^) compared to the low-pH structures (Table 2). As expected from the face-to-face and face-to-back arrangements of the monomers in the low-pH and neutral-pH structures, respectively, a rotation of ∼180° (plus a translation of 19–25 Å) is required to align the non-superposed monomers B, resulting in totally different monomer–monomer interfacial interactions between the two classes of structures (Table 2)

In a very interesting observation, even though monomers A and B in 2Z16 are also arranged in face-to-back fashion as observed in 1EA3, a 14.6 Å translation of monomer B along the A–B monomer interface has abolished nearly all contacts between the two monomers, including the electrostatic interactions seen in 1EA3 (*vide supra*) resulting in a very small buried interface of 423 Å^2^ (Table 2).

Although the pH of crystallization of 3MD2 and 2Z16 are not known we speculate based on their oligomerization state in the crystal that they might have been crystallized at low and neutral pHs, respectively. We note that two other M1 structures reported at neutral pH and low pH (described in publications but without PDB codes) also oligomerize as monomers and dimers, respectively [4], [15].

### Crystal packing and M1 Oligomerization

**Figure 3:**
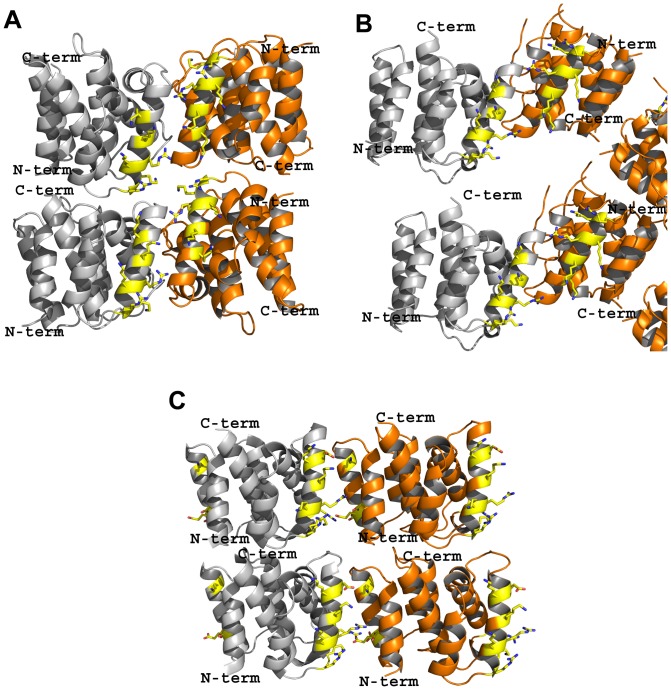
Packing (stacking) arrangement of the M1 molecules (in ribbon diagrams). Monomers A and B are colored grey and orange, respectively. **A.** A dimer of 1AA7 stacked on top of another dimer to form a pseudo-tetramer. A similar arrangement is observed in 3MD2. **B.** A pseudo-tetramer appears to form similarly in 4PUS, however, the two stacked dimers do not interact with each other. **C.** Arrangement of monomers in 1EA3 to form a pseudo-tetramer.

The crystal packing in the dimer 1AA7 structure or the monomer 1EA3 structure have been used to propose several structural models for M1 oligomerization into a polymer that is required to stabilize the virus particles as well as interact with vRNP [4], [12], [14], [15]. In 4PUS, 1AA7 and 3MD2, the dimers stack one on top of another (bottom of one dimer to top of another dimer) to form a “pseudo-tetramer” structure. Sha & Luo were the first to propose a M1 polymerization using the pseudo-tetramer in 1AA7 (Fig. 3A) [12]. Interestingly, the stacking interaction in 4PUS is very loose due to *2_1_* symmetry-related molecules preventing the stacking dimers from coming close together (Fig. 3B).

Similar to the M1 dimer structures, monomers A and B in the neutral-pH 1EA3 structure also form similar bottom-to-top stacking interactions with other monomers, resulting in a pseudo-tetrameric structure (Fig. 3C). The pseudo-tetrameric packing in 2Z16 is quite different as instead of top-to-bottom stacking interaction, the molecules are rather arranged side-to-top. A detailed description of the stacking interactions in low-pH and neutral-pH structures have been described elsewhere [15]. Arzt *et al*. using the pseudo-tetramer observed with 1EA3, proposed an alternate mechanism of M1 oligomerization to form a polymer that involves face-to-back electrostatic interactions between adjacent M1 monomers [4], [14]. In support of the model, the authors pointed to a negative stain electron microscopy study of the M1 layer within the virus that showed no internal symmetry [26], [27], consistent with a monomeric building block. They also proposed that M1 binds the membrane with the positively charged patch of residues [4], [5], [9], and not through hydrophobic interactions as previously suggested by Sha *et al*. [12]. Xie et al., using computational protein–protein docking techniques along with the 1EA2 structure, have also proposed a model in which adjacent M1 N^1–165^ domains may be stacked against one another so as to maximize the electrostatic interaction of the NLS region and nearby basic residues with a cluster of negatively-charged residues of the opposite M1 face and also the M1–M1 steric interactions [10]. This model may be more representative of M1 in its biological environment because it relies only on electrostatic and shape complementarity rather than crystal packing forces to obtain tetrameric structures.

## Discussion

### M1 can access several structural conformations and oligomeric states

Arzt *et al*. using the neutral-pH 1EA3 structure suggested that M1 exists as a monomeric species in solution [14], which is consistent with their gel filtration and small angle neutron scattering data. This conclusion contrasts with an earlier finding by Sha *et al*. that show both truncated and full-length M1 migrate as dimers in gel filtration experiments at low and neutral pHs [12]. A more recent study using gel filtration and sedimentation velocity analytical ultracentrifugation found intact M1 in dimeric and higher oligomeric states at neutral pH and in only dimeric form at low pH [2]. The truncated M1 was also reported to exist as monomer and higher oligomers at neutral pH, but only a monomer at low pH [2]. Zhirnov also found that M1 extracted from M1–vRNP complexes at an acidic pH exhibits monomeric form and does not aggregate following pH neutralization [13]. Using sedimentation velocity analytical ultracentrifugation, we also found the truncated M1 protein to exist in monomeric form at acidic pH. The differing accounts of M1 oligomeric state in solution probably indicate that different oligomeric states may exist in dynamic equilibrium. The “cooperative” free energy, which is the difference in energy of monomer-to-dimer-to-higher oligomer association, may be very small allowing easy conversion from one form to another with subtle changes in pH, ionic strength, etc. The crystallization results of the N-terminal domain M1 also seem to reflect the monomer-dimer association state in solution, which even at similar pH condition shows a propensity to differ in the arrangement and stability of the monomer-monomer association. These observations suggest that M1 is relatively flexible in its oligomeric state in response to external stimuli, which could be a realistic reflection of its biological nature. 

Based on the structural analysis, it appears that the oligomeric state and/or monomer–monomer arrangements of the N-terminal domain of M1 at specific pH are driven by hydrophobic interactions at low pH and electrostatic interactions at neutral pH. At neutral pH, the basic and acidic residues that are located either at the face or back of the monomer should predominantly be ionized, thus driving monomers to associate with each other through electrostatic interactions (Table 2). As the pH is decreased, the acidic residues become increasingly neutralized, which should weaken the electrostatic interaction, disrupting the face-to-back contacts. We also note that Arg134 and Lys98 from subunit A are in close proximity to Lys35 and Lys21 from subunit B, respectively, in the face-to-back orientation at neutral pH (Fig. 2D), which should convert to repulsive interactions between the two monomers as the nearby acidic residues become neutralized and facilitate the dissociation of the monomers. Subsequently the monomers reorient to form a face-to-face dimeric structure driven mainly by hydrophobic interactions that arise mostly between the symmetry-related helices H6. The basic NLS seems not to contribute to the dimer formation, consistent with the report that an NLS knockout M1 mutant (the basic NLS residues were replaced with alanines) still forms a dimer at low pH with identical structure as 1AA7 [4].

### Multi-conformational and multi-oligomeric state of M1 is consistent with its functions

M1 stabilizes the virus particles at neutral pH by forming a matrix layer under the viral envelope through self-polymerization and interactions with the lipid membrane and vRNP. Acidification of the endosomes containing the viral particles causes depolymerization of M1, as well as dissociation from the membrane and vRNP [18], [28]-[31], allowing vRNP to enter the nucleus. The process of M1 dissociation from the membrane and vRNP is shown to be preceded by a conformational change in the protein [19]. This underscores the multifunctional activities of M1 that may necessitate the ability to adopt different conformations depending on the environment. The recently reported study by Fontana & Stevens using cryo-electron microscopy and cryo-electron tomography showed most virions at neutral pH have an M1 matrix layer that disappeared in 50% of the virions at low pH [19]. For the virions that retained the M1 layer at low pH, significant numbers showed extended patches of altered M1 structure, which the authors suggested is a manifestation of a conformational change in M1 and a prelude to dissociation of the matrix layer [19].

**Figure 4:**
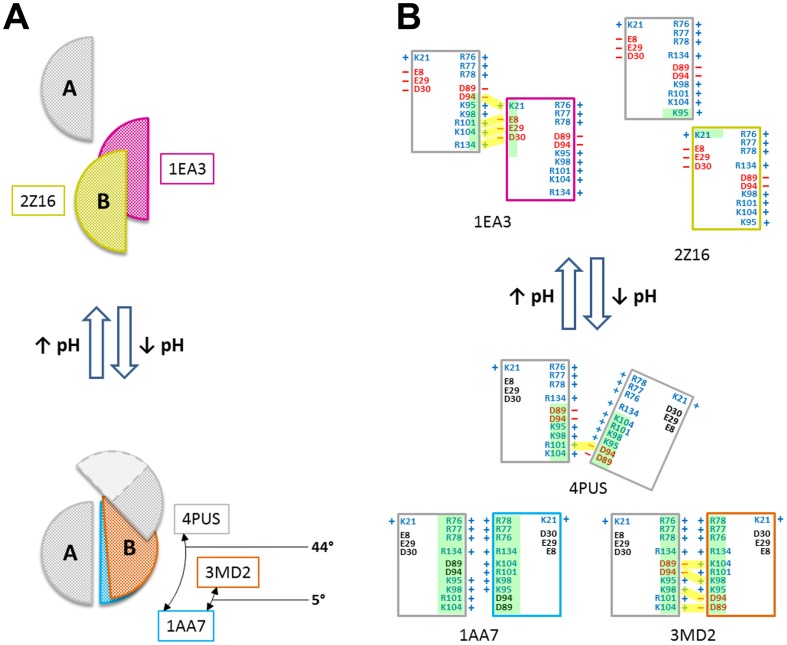
Schematic representation of the proposed multiple conformational transitions in M1 structures. **A**. Cartoon diagrams illustrating the oligomeric state of the M1 crystal structures, including 4PUS (grey), 1AA7 (blue), 3MD2 (red), 1EA3 (magenta) and 2Z16 (yellowish-green). Structures at neutral pH (top) are monomeric and arranged face-to-back; those at acidic pH (bottom) are dimeric and arranged face-to-face. Disorder in the Safo (4PUS) structure is indicated by a light-colored region bounded by a dashed line. **B**. Cartoon illustrating the interfacial regions of 4PUS (grey), 1AA7 (blue), 3MD2 (red), 1EA3 (magenta) and 2Z16 (yellowish-green). Individual M1 chains (consisting of two four-helix bundles separated by a linker) are represented as rectangles. Each crystal structure is represented by an ‘A’ chain (leftmost rectangle; grey border) and a ‘B’ chain (rightmost rectangle). The acidic (red) and basic (blue) residues at the interfacial regions are labeled and may be grouped into five clusters (K21; E8, E29 and D30; R76, R77 and R78; D89, D94, K95, K98, R101 and K104; R134) based on their proximity to one another on the M1 surface. Salt bridges are represented by yellow rectangles joining opposite charges. Note that the salt-bridge interactions depicted in Liu (3MD2) are>3.7 Å. Green transparent boxes within the rectangles represent the relative amount of shared interfacial surface area (Table 2).

We propose that 1EA3 and 1AA7 may represent two “boundary” structural arrangements of M1 at neutral and low pH, respectively; with the other structures representing “intermediate” stable transitions or snapshots (Fig. 4). The observed M1 matrix structure found in the virions at neutral pH can be described by 1EA3 monomeric “building blocks” that are driven to polymerize through electrostatic interactions in a face-to-back fashion (Fig. 2D). 2Z16, which shows significant monomer–monomer interface sliding may characterize a structural snapshot that precedes dissociation of the M1 layer (formed by 1EA3) as the medium begins to change from neutral to acidic (Figs. 4). 2Z16 could also represent an intermediate structure involved in the assembly of the matrix. The low-pH structures that are dimers may describe the dissociated M1 from the polymer as well as from the membrane and/or vRNP that occur at acidic pH (Fig. 4), consistent with the finding that M1 oligomers dissociate into dimers at low pH [2]. Although we propose 4PUS and 3MD2 as possible structural intermediates during the formation of 1AA7 from the neutral pH structures, it is also conceivable that any of the low-pH structures could precede or follow one another during the monomeric ↔ dimeric structure transition.

Another important function of M1 is to translocate into the nucleus at a later stage in the viral cycle to mediate interaction between NEP/NS2 for export of the newly synthesized vRNP to the cytoplasm to form new particles [30], [32]–[37]. In contrast to earlier studies that suggest M1 binds to vRNP with its NLS [30], [36], later studies however suggest M1 binding to vRNP with its C^166–252^-domain [4], [5], while the NLS is used for interacting with a cluster of glutamate residues and an exposed Trp78 of NEP/NS2 [3]. M1 could assume a dimeric structure or monomeric structure where the basic residues are exposed (*vide infra*) to facilitate nucleus translocation. Considering the fact that the pH of the nucleus may be neutral, M1 may associate as monomers to bind NEP/NS2 and vRNP in the nucleus.

The disorder of the first 80 N-terminal amino acids in monomer B of 4PUS is very unique because other M1 structures do not have this disorder. Fontana & Steven showed that virions are characterized by multiple conformational states of M1 layer and dissociation of the M1 layer is preceded by a conformational change in the protein [19], which they proposed could be due to disorganization of the C^166–252^-domain because the authors did not observe any apparent structural differences between the low-pH and neutral-pH M1 N^1–165^-domain monomers. Given that there is no crystal packing explanation, the observed disorder in 4PUS may also in part be a prelude for such conformational change proposed by Fontana and Steven [19]. Sha *et al*. [12] has also suggested a conformational change in the protein that would expose buried hydrophobic residues for viral membrane binding. The observed disorder in 4PUS could also be a precursor for such conformational change. Nonetheless, it should be noted that other studies suggest that the interaction between M1 and the membrane is not hydrophobic but rather electrostatic via the NLS [9]. It is also possible that a conformational change in the N^1–165^-domain is a structural requirement to transition among different M1 structures.

## Conclusions

We report a unique dimer structure of M1 at low pH of crystallization that is distinct from previously reported structures at similar conditions. One of the monomers in the new structure shows considerable and inherent disorder that could be physiologically relevant, e.g. in the destabilization of the matrix layer among other functions. Through rigorous analysis of all available M1 structures that were classified into monomeric and dimeric structures, we show that within the two classes, the structures could differ both in monomer structure and monomer–monomer arrangements. Biochemical evidence also suggests several oligomeric and multi-conformational states to exist in solution. These findings indicate several accessible variations in the M1 monomer and/or dimer and/or oligomeric structures that occur on a continuum of pH (and/or other factors) that may be necessary for the multifunctional activities of M1, consistent with an earlier suggestion that a pH-dependent structural change of M1 is a key factor that regulates the interactions of M1 with other viral components [19], [28].
